# Optimizing Processing Technology of *Cornus officinalis*: Based on Anti-Fibrotic Activity

**DOI:** 10.3389/fnut.2022.807071

**Published:** 2022-05-03

**Authors:** Xin Han, Chuan Ding, Yan Ning, QiYuan Shan, Minjie Niu, Hao Cai, Peng Xu, Gang Cao

**Affiliations:** ^1^School of Pharmacy, Zhejiang Chinese Medical University, Hangzhou, China; ^2^School of Pharmacy, Nanjing University of Chinese Medicine, Nanjing, China; ^3^The Third Affiliated Hospital of Zhejiang Chinese Medical University, Hangzhou, China

**Keywords:** *Cornus officinalis*, fibrosis, wine steamed, processing technology, composition difference

## Abstract

*Cornus officinalis*, a kind of edible herbal medicine, has been widely used in the protection of liver and kidney due to its medicinal and nutritional effect. Its anti-inflammatory, anti-tumor, and anti-oxidant activities can be enhanced by wine-steamed (WS) processing. Based on the activations of hepatic stellate cells-T6 (HSC-T6) and HK-2, our study used single-factor plus orthogonal design to investigate the anti-fibrosis of *C. officinalis* processed with steamed (S), high-pressure steamed (HPS), WS, high-pressure wine-steamed (HPWS), wine-dipped (WD), and wine-fried (WF). The chemical constituents in processed *C. officinalis* with higher anti-fibrotic activities were detected by ultra-high performance liquid chromatography coupled with hybrid triple quadrupole time-of-flight mass spectrometry (UHPLC-Q-TOF-MS/MS). Results showed that *C. officinalis* with HPWS significantly inhibited the activations of HSC-T6 and HK-2. Moreover, compounds in *C. officinalis* with HPWS were obtained *via* UHPLC-Q-TOF-MS/MS, indicating that 27 components were changed compared with raw *C. officinalis*. These results demonstrated that HPWS is the optimal processing technology for anti-fibrosis of *C. officinalis*.

## Graphical Abstract

The processing technology of C. officinalis was optimized in terms of its anti-fibrotic activity. Differences in its chemical composition after the preparation were detected by UHPLC-Q-TOF-MS/MS. These results provide material basis for further exploring the role of *C. officinalis* in liver and kidney protection.
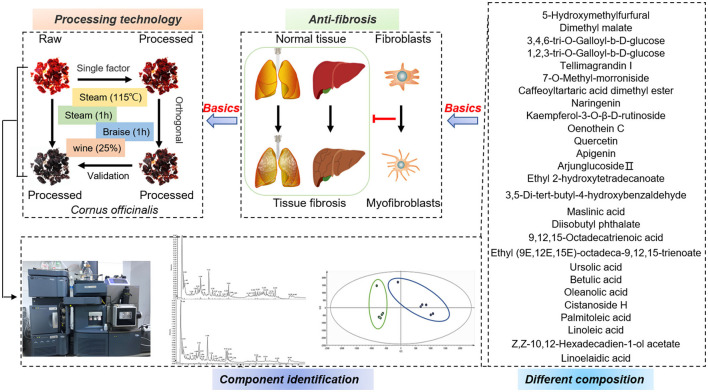

## Introduction

*Cornus officinalis* (*C. officinalis*), the dry mature fruit of *C. officinalis* Siebold & Zucc, is redefined as a class of herb and edible plant and has been commonly used in traditional Chinese medicine (TCM) (1). *C. officinalis* with mild warm nature, belongs to the meridians of the liver and kidney according to TCM theory; thus, it is commonly used in the prevention and treatments of liver and kidney diseases (2). Moreover, it can be found in foodstuff, such as medicinal dishes, healthcare products, and drinks due to its various pharmacological activities, including anti-inflammatory, antioxidant, and anti-apoptotic (3). To date, about 305 components have been isolated and identified from *C. officinalis*, including iridoids, alkaloids, polysaccharides, flavones, organic acid, essential oils, and terpenoids (1). Among these compounds, loganin and morroniside are active ingredients in *C. officinalis* and could alleviate osteoarthritis in mice by inhibiting pyroptosis and NF-kappaB activity (4, 5). Furthermore, morroniside could ameliorate neuropathic pain through the regulation of glucagon-like peptide-1 (GLP-1) receptors. 5-hydroxymethylfurfural (5-HMF), which are mainly isolated from processed *C. officinalis*, could prevent human umbilical vein endothelial cells (HUVECs) from oxidative stress induced by glucose (6). However, the compounds in *C. officinalis* may change with processing and in turn affect pharmacological activities of *C. officinalis*. In traditional crafts of China, *C. officinalis* were often processed with fried, steamed, wined, fated, salted, and others, to meet different clinical effects. For example, wined *C. officinalis* is most commonly used in clinical preparations, such as the Liuwei Dihuang pills in China, which has better effects in nourishing the liver and kidney compared to raw (R) *C. officinalis*. However, there is no unified requirement for steamed, braised times and wine amount, all of which could lead to the difference in the compositions and activities of *C. officinalis*. Therefore, the present study aimed to optimize the processing technology of *C. officinalis* through the single-factor method plus orthogonal experiment design based on the anti-fibrotic activities of *C. officinalis*.

Tissue fibrosis, characterized by excessive deposition of extracellular matrix (ECM), is the outcome of chronic tissue damage, leading to the formation of scar tissue, if without treatments, fibrosis will promote organ dysfunction and even failure (7). The ECM is mainly derived from proliferative and fibrogenic myofibroblasts, which are fibroblast-like cells with contractile properties (8, 9). Activated hepatic stellate cells (HSC-T6) are the main source of myofibroblasts and serve as a key driver of hepatic fibrosis in liver injury (9). In normal liver, HSC-T6 are in quiescent state and involved in the storage of vitamin A (10). During liver injury, quiescent HSC-T6 are activated, which indicated by the high expression of alpha smooth muscle actin (α-SMA) and excessive deposition of ECM (11). Similar to HSC-T6, the human proximal tubular epithelial cell (HK-2) cells play a key role in tubulointerstitial fibrosis (TIF), which is the final result of chronic kidney disease and closely related to the degeneration of renal function (12). Moreover, epithelial mesenchymal transition (EMT) is the main pathogenesis of the renal fibrosis and can transform differentiated epithelial cells into myofibroblasts, which are also characterized by the high expression of α-SMA (13, 14). During fibrogenesis, EMT may be driven by transforming growth factor β (TGF-β), which is the most important pro-fibrotic growth factors (15). Apart from these, TGF-β is regarded as a common vital switch for fibrosis in tissue or organs in response to chronic injuries (16).

In summary, we will evaluate the effects of *C. officinalis* on anti-fibrosis *via* the expressions of α-SMA in TGF-β induced-HSC-T6 and HK-2 cells, with the goal of exploring the protective effect of *C. officinalis* on the liver and kidney, and finally select the processing technology with higher anti-fibrotic activity. Moreover, the changes of chemical constituents in processed *C. officinalis* will be detected by ultra-high performance liquid chromatography coupled with hybrid triple quadrupole time-of-flight mass spectrometry (UHPLC-Q-TOF-MS/MS).

## Materials and Methods

### Plant Materials and Processing Technology

The samples of *C. officinalis* were selected from the PanAn City of Zhejiang Province and identified by Professor Jianwei Chen of the Department of Chinese Medicine Identification, School of Pharmacy, Nanjing University of Chinese Medicine. According to single-factor experiments, *C. officinalis* samples were steamed at different times (1, 2, 4, 6, and 8 h), different temperatures (100, 105, 110, 115, 120, and 125°C) and then dried at 60°C, respectively. Next, the samples were braised at different times (0.5, 1, 2, and 4 h) with different dosages of rice wine (w/w) (15, 20, 25, 30, and 40%), respectively. These samples were then steamed again for 1 h at 105°C and then finally dried at 60°C. The orthogonal experimental design was based on the results of single-factor experiments as discussed in the following: The *C. officinalis* samples were processed with three factors (steamed times, steamed temperatures, and braised times) at different dosages of rice wine in line with L^9^ (3^4^) design orthogonal table shown in S1, S3, and S5, respectively. Moreover, the optimal parameters of *C. officinalis* with high-pressure wine steamed (HPWS) were identified with steamed for 1 h at 115°C, braised time of 1 h, and rice wine dosage (w/w) at 25%. The HPS was obtained with processed for 1 h at 125°C. Additionally, *C. officinalis* could be processed with wine-dipped (WD) and wine fried (WF). *Cornus officinalis* that steamed for 1 h was named as S.

### Extraction Preparation

The processed *C. officinalis* (both R and processed) was crushed and sifted through 16-mesh screen. The powder (5 g) was refluxed with 90% ethanol (50 ml) twice and filtered with four layers of gauze. The filtrates were collected and combined. Next, the filtrates were transformed into freeze-dried powder through vacuum concentration and lyophilization.

### UHPLC-Q-TOF-MS/MS Analysis

The LC–MS/MS analysis were performed using an UHPLC (Shimadzu LC-30AD, Japan) coupled with a Triple TOF 5600 Plus System (AB Sciex, USA) (17, 18). The parameters that were followed were as follows: Column temperature was 30°C; mobile phase was acetonitrile (B) and 0.1% formic acid in water (A); flow rate was 0.3 ml/min; and the injection volume was 3 μl. The gradient elution procedure was as follows: 0–3.0 min, 5–20% B; 3.0–7.0 min, 20–80% B; 7.0–30 min, 80–90% B; 30–32 min, 90–5% B; 30–32 min, B was maintained at 5%. For UHPLC separation, the samples were analyzed with a 2.1 mm × 100 mm ZORBAX Extend-C18 1.8 μm column (Agilent, USA). The conditions in both electrospray ionization (ESI), positive and negative modes were set as follows: The ion source temperature was 550°C, IonSpray voltage was 5,500–5,500 V, auxiliary spray gas was nitrogen, Ion Source Gas1 (Gas1) was 55 psi, Ion Source Gas2 (Gas2) was 55 psi, curtain gas (CUR) was 35 psi, declustering potential (DP) was 60 V, and collision energy was 30 V. The scanned ranges of TOF–MS and TOF–MS/MS were 100–2,000 and 50–1,000 Da, respectively.

### Cell Culture and Treatment

The HSC-T6 and HK-2 cells were cultured in Dulbecco's modified Eagle medium (DMEM) (Abcam, UK) and Dulbecco's Modified Eagle Media DMEM/F12 (Abcam, UK), respectively, supplemented with 100 mg/ml streptomycin, 100 U/ml penicillin, and 10% fetal bovine serum (FBS) (Abcam, UK) under 5% CO_2_ at 37°C. When establishing liver fibrosis *in vitro*, HSC-T6 and HK-2 cells were cultured in six-well plates and treated with TGF-β (PeproTech, USA), respectively. To detect the effects of *C. officinalis* on anti-fibrosis, cells were treated with extracts derived from *C. officinalis* (both R and processed).

### Western Blot Analysis

The cells were lysed using radio-immunoprecipitation assay (RIPA) buffer supplemented with 1% phenylmethanesulfonyl fluoride (PMSF) (Solarbio, Beijing, China) and then quantified by BCA protein assay kit (Beyotime, Shanghai, China). The equivalent protein samples were separated by sodium dodecyl sulfate–polyacrylamid gel electrophoresis (SDS–PAGE) and then transferred to polyvinylidene fluoride (PVDF) membranes (GE, Freiburg, Germany). The membranes were blocked with 5% skimmed milk for 1 h at room temperature and incubated with anti-α-SMA antibody (Abcam, UK) overnight at 4°C. The membranes were incubated with horse radish peroxidase (HRP)-conjugated secondary antibodies for 1 h at room temperature and visualized with ECL Reagent (Beyotime, Shanghai, China). Finally, the membranes were stripped and probed with GAPDH (loading control). The intensities of bands were quantified by Quantity One software (Bio-Rad, Hercules, CA, USA).

### Immunocytochemistry

The HSC-T6 cells were cultured in six-well plates and fixed with 4% paraformaldehyde, permeabilized in 0.1% Triton X-100, and then incubated with rabbit polyclonal anti-α*-*SMA (1:100 dilution) (Abcam, UK) at 4°C overnight. The cells were then incubated with goat anti-rabbit IgG H&L (Alexa Fluor^®^ 488, 1:200 dilution), after which the cell nuclei were stained with DAPI (Beyotime, Shanghai, China). Finally, the images were observed and analyzed with a Nikon TI-E fluorescence microscope.

### Statistical Analysis

The data in the experiments were present as mean ± SD. The comparison of the results was evaluated by GraphPad Prism program (Graphpad Software, Inc., San Diego, CA, USA) with one-way analysis of variance (ANOVA) and Tukey's multiple comparison tests. Statistical significance between groups was considered with a *p* < 0.05. The difference analyses were detected by MarkerView™ and *t*-test. The original data were imported into MarkerView™ and statistical analysis *via t*-test. The *t*-test was employed to identify significant differences among processed products. Here, *p* < 0.05 was considered a significant difference, and a *t* > 0 indicated an increase in ingredient contents.

## Results and Discussion

### Evaluation of *C. officinalis* (R) on Anti-fibrosis

**Figure 1:**
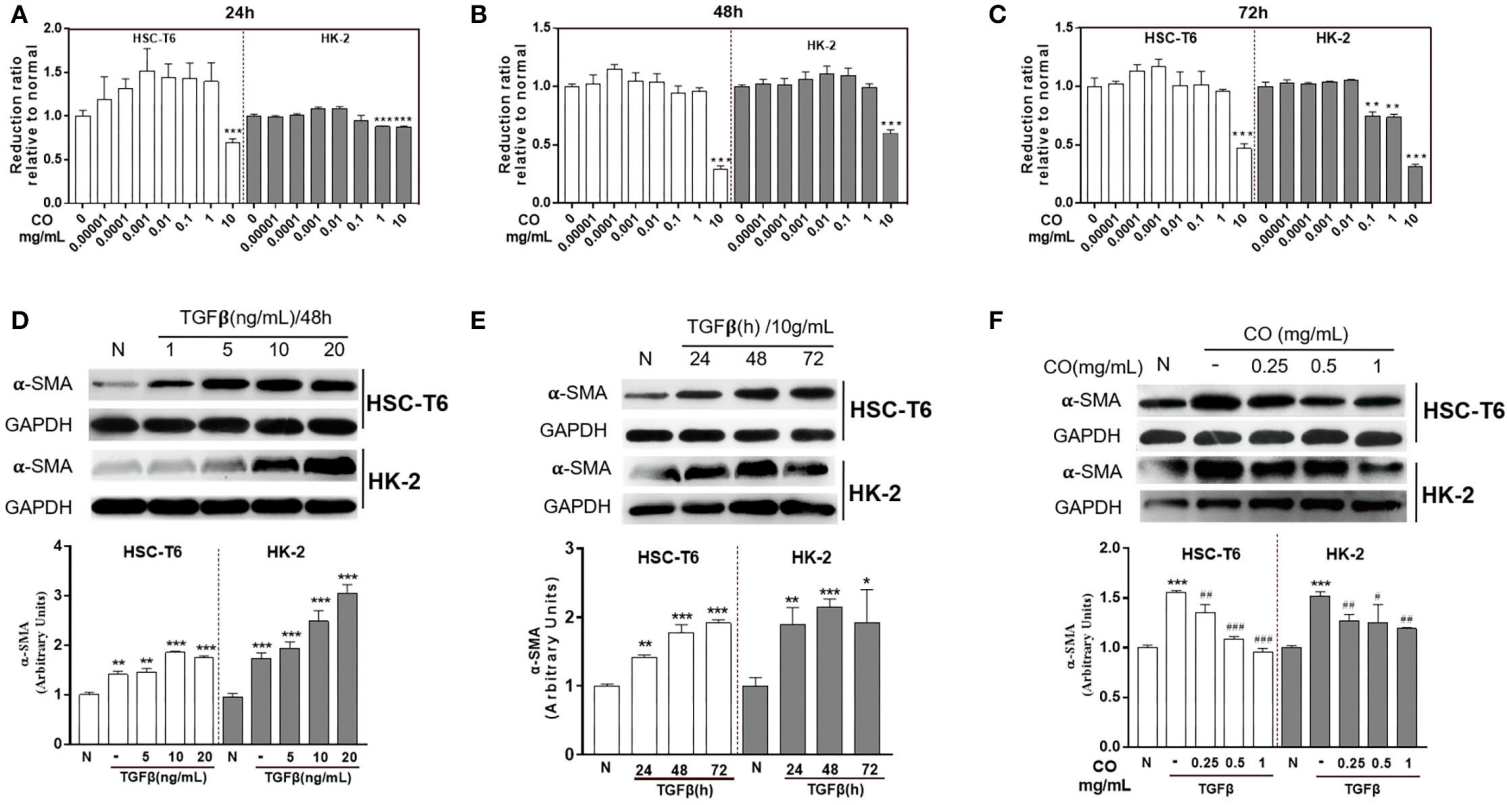
Pharmacodynamic evaluation of *C. officinalis*. The cytotoxicity of *C. officinalis* on HSC-T6 and HK2 cells for 24 **(A)**, 48 **(B)**, and 72 h **(C)**. HSC-T6 and HK2 cells were activated by TGF-β with different concentrations **(D)** and for different times **(E)**. **(F)** The effects of *C. officinalis* on activated HSC-T6and HK2 cells. Data are represented as means ± SEM. *p ≤ 0.05, **p ≤ 0.01, and ***p ≤ 0.001 vs. the control group; ^#^*p* ≤ 0.05, ^#^*p* ≤ 0.01, and ^###^*p* ≤ 0.001 vs. the TGF-β group.

The cytotoxicity of *C. officinalis* (R) on HSC-T6 and HK-2 cells were evaluation by CCK8, which is a widely method in the detection of cytotoxicity and drug sensitivity. In this study, HSC-T6 and HK-2 cells were treated with different doses of *C. officinalis* (0–10 mg/ml) for 24, 48, and 72 h. Results showed that *C. officinalis* (R) at a dose range of 0–1 mg/ml had little cytotoxicity on HSC-T6, while the sample at a dose of 10 mg/ml significantly inhibited the survival of cells compared with the normal sample at 24, 48, and 72 h. For HK-2 cells, the minimal cytotoxicity of *C. officinalis* at a dose range of 0–1 mg/ml was observed only at 48 h and was not observed at 24 and 72 h (Figures 1A–C, respectively). Therefore, we chose 48 h for the follow-up experiments.

To establish a preferable fibrosis model *in vitro*, HSC-T6 and HK-2 cells were treated with different concentrations of TGF-β at different times. The results showed that TGF-β at 1, 5, 10, and 20 ng/ml activated HSC-T6 and HK-2 cells, which manifested by the higher expressions of α-SMA compared to the normal group (Figure 1D), moreover, TGF-β at different times 24, 48, and 72 h also up-regulated the expressions of α-SMA in HSC-T6 and HK-2 cells (Figure 1E). On comparing the results, TGF-β (10 ng/ml) at 48 h was used to activate the cells. Moreover, Figure 1D showed that *C. officinalis* (R) at different doses (0.25, 0.5, and 1 mg/ml) inhibited the expressions of α-SMA increased by TGF-β in HSC-T6 and HK-2 cells, with the best effect identified at a dose of 1 mg/ml (Figure 1F).

In TCM, *C. officinalis* was often used in replenishing the liver and kidney due to its tonic effect (1). Furthermore, modern pharmacology indicated that *C. officinalis* showed low toxicity on cells only at high concentrations (19), as confirmed by the results of CCK8. The present study also provided a theoretical basis for further research on liver- and kidney-related diseases. In liver and kidney fibrosis, persistent or dysregulated fibrogenic reactions may hamper regeneration and promote dysfunction (20), which could ultimately raise susceptibility to organ failure and death (21). During these processes, TGF-β contributes to a fibrogenic phenotype by activating fibroblasts cells (20), including HSC-T6 and HK-2 cells. Taking all these elements into account, the present study aimed to develop a therapeutic implementation of anti-TGF-β approaches. The results showed that *C. officinalis* down-regulated the TGF-β-induced expression of α-SMA and provided a direction for us to optimize the processing technology of *C. officinalis* with higher anti-fibrotic activity.

### Effects of Processed *C. officinalis* on Anti-fibrosis Changes With Univariate Elements

**Figure 2:**
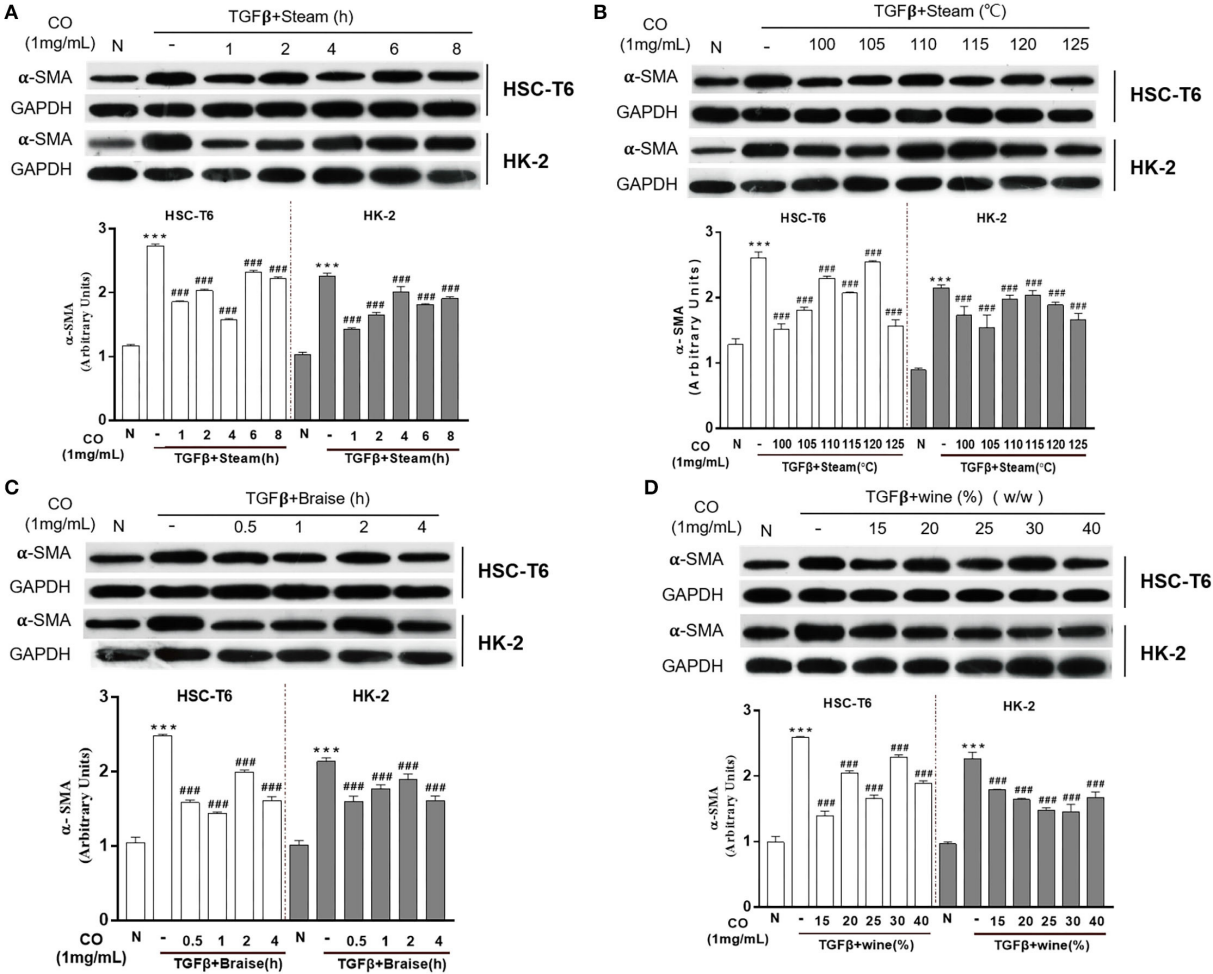
Effects of processed *C. officinalis* on anti-fibrosis changes with univariate elements. Anti-fibrosis activities of the *C. officinalis* samples steamed at different times **(A)**, different temperatures **(B)**, braised at different times **(C)**, and with different rice wine dosages (w/w) **(D)**. Data are represented as means ± SEM. ***p ≤ 0.001 vs. the control group. ^###^*p* < 0.01 vs. the TGF-β group.

The processing methods of *C. officinalis* found in the Pharmacopeia of the People's Republic of China (ChP) including wine-steamed (WS) and wine-braised processing. To identify and optimize the processing technology with the best anti-fibrosis activity, single-factor experiment was used in this study. The results showed that *C. officinalis* steamed at different times (1, 2, 4, 6, and 8 h) or temperatures (100, 105, 110, 115, 120, and 125°C) inhibited the expressions of α-SMA induced by TGF-β both in HSC-T6 and HK-2 cells. Among these, for HSCs cells, the anti-liver fibrosis activity of *C. officinalis* steamed for 4 h at 100°C was the best, while steamed at a temperature of 105°C for 1 h had the best anti-fibrosis effects on HK-2 cells (Figures 2A,B, respectively). Furthermore, braised *C. officinalis* for 0.5, 1, 2, and 4 h inhibited TGF-β-induced over-expressions of α-SMA in HSC-T6 and HK-2 cells, especially at 1 h in HSC-T6 and 4 h in HK2 cells (Figure 2C). *C. officinalis* processed with different dosages of rice wine (w/w) also showed different degrees of anti-fibrosis activities, especially 15% in HSC-T6 and 25% in HK-2 cells (Figure 2D).

Wined *C. officinalis* is a traditional and common processing method that applied until now. However, different indexes of evaluation are accompanied by varying wine processing techniques. Thus, this research aimed to explore the suitable wined technology of *C. officinalis* based on the anti-fibrosis synergistic effects produced by each technique. The results showed that the anti-fibrosis effect of *C. officinalis* was negatively correlated with the expression of α-SMA. Thus, the data were non-negatively analyzed, which are discussed as follows: Xij = max (X1j, X2j, …, Xnj) – Xij/max (X1j, X2j, …, Xnj) – min (X1j, X2j, …, Xnj) + 1; i = 1, 2, …, n, j = 1, 2, …, n). Moreover, the expressions of α-SMA in HSC-T6 and HK-2 cells were used as indicators for comprehensive scoring, each with a weight of 0.5. The composite score was calculated using the following formula: Xij (HK-2) × 0.5 + Xij (HSC-T6) × 0.5. The results showed that *C. officinalis* processed with the following parameters: steamed time of 1 h, steamed temperatures of 105 and 125°C, braised times of 0.5 and 4 h, and rice wine dosage (w/w) at 25%, respectively, had the best inhibitory effects on activated HSC-T6 and HK-2; thus, laying the foundation for the subsequent orthogonal experiments. However, considering the time benefit, the term of orthogonal experiment was finally determined as follows: The steamed times were 1, 2, and 3 h; steamed temperatures were 105, 115, and 125°C; braised times were 0.5, 1, and 1.5 h; and rice wine dosages (w/w) were 25, 30, and 35%.

### Optimization of the Processing Technology With Orthogonal Test

**Figure 3:**
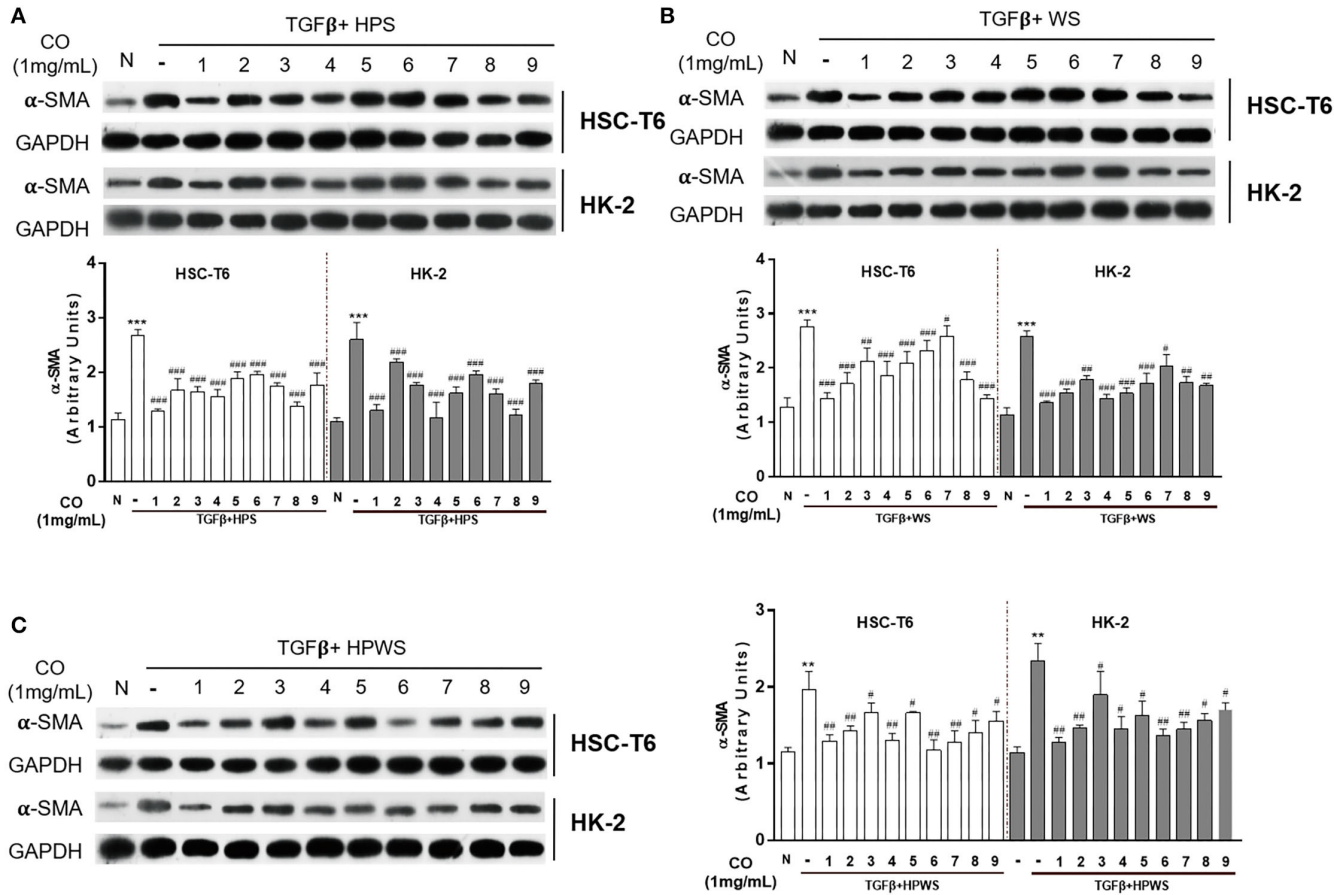
Optimization of the processing technology with orthogonal test. Anti-fibrosis activities of the *C. officinalis* samples processed with HPS **(A)**, WS **(B)**, and HPWS **(C)** technologies. Data are represented as means ± SEM. **p ≤ 0.01 and ***p ≤ 0.001 vs. the control group. ^#^*p* < 0.05, ^##^*p* < 0.01, and ^###^*p* < 0.01 vs. the TGF-β group.

In accordance with the terms of the single-factor experiments, the processing parameters were optimized with an orthogonal L^9^ (3^4^) test design. Our results showed that *C. officinalis* processed with three levels of two factors (steamed times and steamed temperatures), as shown in Supplementary Table S1, inhibited the expressions of α-SMA in HSC-T6 and HK-2 induced by TGF-β (Figure 3A). The composite score of the anti-fibrosis effects was calculated with non-negative analysis, and the results indicated that the maximum was 1.92 (Supplementary Table S1). Furthermore, in selecting the better processing term, the values of K and R are shown in Supplementary Table S1, and the variance analysis results are shown in Supplementary Table S2. These results showed that *C. officinalis* steamed (HPS) showed optimum anti-fibrosis effect. The craft conditions of *C. officinalis* with WS were also optimized with an orthogonal design (Supplementary Table S3), and its anti-fibrosis effects on both HSC-T6 and HK-2 were evaluated *via* the expressions of α-SMA induced by TGF-β (Figure 3B). The results indicated that *C. officinalis* with WS inhibited the activation of myoblasts, which was mainly manifested by the decrease in levels of α-SMA (Figure 3B). Combined with the results of variance analysis (Supplementary Table S4) and the values of *K* and *R* in Supplementary Table S3, the optimal processing term with anti-fibrosis effects were as follows: steamed time of 2 h, braised time of 0.5 h, and rice wine dosage (w/w) of 30%. Similarly, steamed HWPS were optimized with orthogonal design (Supplementary Table S5), variance analysis (Supplementary Table S6), and the expression of α-SMA (Figure 3C).

Due to the fact that steamed time, steamed temperature, braised time, and rice wine dosage are vital criteria in the processing of *C. officinalis* (22), processed *C. officinalis* using different technologies have varying antidiabetic effects (23). Besides, the results of our study showed that the *C. officinalis* had different inhibitory effects on activated fibrosis cells. Therefore, optimization of processing criteria is the essential step in screening the technology with the optimal anti-fibrosis effect. Studies have shown that the orthogonal test design is a common method for the optimization of experimental conditions (24, 25). Based on these, the current study used the orthogonal L^9^ (3^4^) test design to detect the anti-fibrosis of *C. officinalis* processed with different factors for the single-factor experiments and obtained several artifacts of *C. officinalis via* S, WS, and HPWS technologies.

### Validation of Processed *C. officinalis* in Terms of Anti-fibrosis Effects

**Figure 4:**
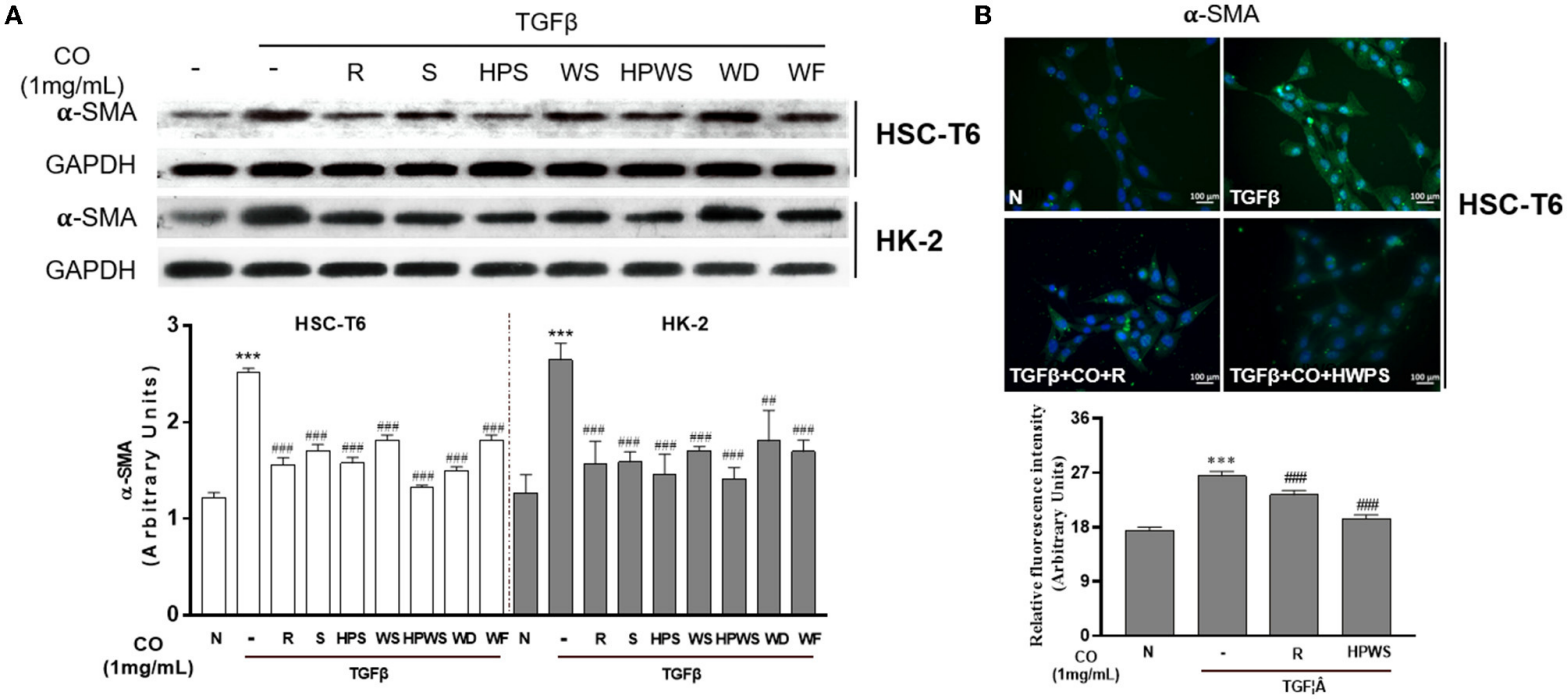
Validation of processed *C. officinalis* in terms of anti-fibrosis activity. The anti-fibrosis activities of all processed *C. officinalis* samples were detected by Western blot analysis **(A)**; the effects of immunofluorescent staining of α-SMA in HSC-T6 treated with *C. officinalis* processed with HPWS present in 400 × magnification **(B)**. Data are represented as means ± SEM. *p ≤ 0.05, **p ≤ 0.01, and ***p ≤ 0.001 vs. the control group. ^#^*p* < 0.05, ^##^*p* < 0.01, and ^###^*p* < 0.01 vs. the TGF-β group.

The optimal processing technologies of processed *C. officinalis* were determined by single-factor plus orthogonal experiments. The processed products of *C. officinalis* were prepared according to the terms of HPWS and the stipulation in Chp, including WD and WF technology. The expressions of α-SMA were detected by western blot to verify the anti-fibrosis activity of all processed products of *C. officinalis*. The results showed that *C. officinalis* (both R and processed products) inhibited the expressions of α-SMA induced by TGF-β in both HSC-T6 and HK-2, especially *C. officinalis* processed with HPWS (Figure 4A). Then, immunofluorescence assay for α-SMA in HSC-T6 was conducted to confirm the anti-fibrosis effect enhanced by *C. officinalis* processed with HPWS. The results also showed that *C. officinalis* inhibited the positive expression of α-SMA (in green) induced by TGF-β, especially the *C. officinalis* processed with HPWS (Figure 4B).

Based on the single-factor, orthogonal tests, variance analysis, and the evaluation of anti-fibrosis activity, *C. officinalis* processed with HPWS showed better anti-fibrosis activity than other processed products of *C. officinalis*. The differences in the anti-fibrosis activities of *C. officinalis* are mainly due to various active components in the *C. officinalis* (26), which are possible to transform with qualitative and quantitative changes during processing. Therefore, exploring changes of composition in *C. officinalis* processed with HPWS can provide material basis for further clarifying the mechanism of *C. officinalis* with HPWS-enhanced anti-hepatic fibrosis.

### Ingredient Identification of *C. officinalis* Processed With HPWS

**Figure 5:**
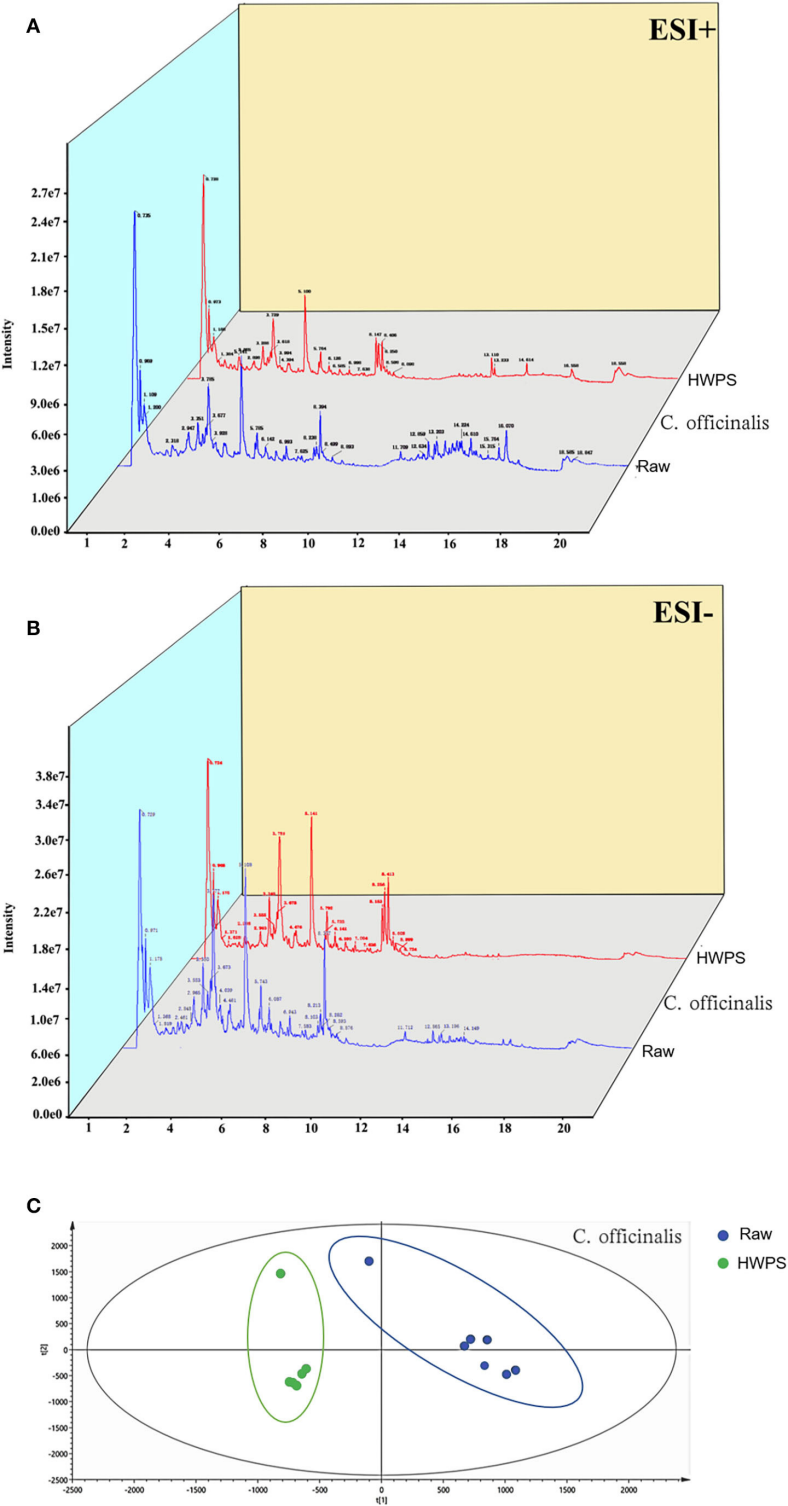
Ingredient identification of *C. officinalis* processed with HPWS. **(A)** Raw sample positive and negative ion pattern diagram. **(B)** The product's positive and negative ion pattern is illustrated. **(C)** PLS-DA score plot.

The analysis of the compounds in *C. officinalis* (both R and HPWS) were identified by ESI positive and negative modes (Figure 5). The chemical name, molecular mass, molecular formula, and molecular structure of components in *C. officinalis* were retrieved and download from the database, after which the accurate mass-to-charge ratio of plasma morphology were calculated in both ESI positive and negative modes. The raw data were imported into the PeakView™ software. All the chemical components were encoded and a new session was established under the XIC Manager template. Then, the first-level data matching was conducted with reference standards, standard mass spectrometric database, and literature according to m/z. The chromatographic peak with the retention time error within 0.2 min and the m/z error within 10 ppm was identified as a unified compound. Further, the identification validation and chromatographic peak attribution were based on molecular structures and secondary fragments of compounds.

As shown in Table 1, 27 components in *C. officinalis* were changed with HPWS, including flavonoids, iridoid glycosides, and organic acids. Moreover, 5-HMF was a typical emerging compound in *C. officinalis* with HPWS. We take quercetin as an example to illustrate the identification procedure: [M – H]^−^ of peak 11 was 301.0354, formula was calculated as C_15_H_10_O_7_ with mass (Da) 302.0427, the main secondary fragment was 121.0305, and neutral loss was 180.0019. After consulting the literature (27) and comparing the results with standards, we confirmed that the compound is quercetin.

**Table 1:** Different chemical composition of the raw and HPWS *C. officinalis* products.

		**Formula**	**Mass (Da)**	**Adduct**	**Extraction**	**Error**	**RT**	***t***	***p***	**Changedirection**
					**Mass (Da)**	**(ppm)**	**(min)**	
1	5-Hydroxymethylfurfural	C_6_H_6_O_3_	126.0317	[M+H]^+^	127.03897	1.5	1.9	3.548359	0.00528	+
2	Dimethyl malate	C_6_H_10_O_5_	162.0528	[M-H]^−^	161.04555	5.2	2.32	−4.381084	0.00138	–
3	3,4,6-tri-O-Galloyl-b-D-glucose	C_27_H_24_O_18_	636.0963	[M-H]^−^	635.08899	4.5	3.87	−2.576338	0.02759	–
4	1,2,3-tri-O-Galloyl-b-D-glucose	C_27_H_24_O_18_	636.0963	[M-H]^−^	635.08899	5.7	3.9	3.263964	0.00852	+
5	Tellimagrandin I	C_34_H_26_O_22_	786.0916	[M-H]^−^	785.0843	5.8	4.54	2.879021	0.01641	+
6	7-O-Methyl-morroniside	C_18_H_28_O_11_	420.1632	[M-H]^−^	419.15589	2.6	4.97	4.730597	0.0008	+
7	Caffeoyltartaric acid dimethyl ester	C_15_H_16_O_9_	340.0794	[M+H]^+^	341.08671	0.1	5.01	5.844058	0.00016	+
8	Naringenin	C_15_H_12_O_5_	272.0685	[M+H]^+^	273.07575	0.9	6.73	4.134988	0.00203	+
9	Kaempferol-3-O-β-D-rutinoside	C_27_H_30_O_15_	594.1585	[M+H]^+^	595.16575	0.7	7.29	3.041758	0.01243	+
10	Oenothein C	C_34_H_24_O_22_	784.0759	[M+H]^+^	785.0832	0.1	7.32	5.861379	0.00016	+
11	Quercetin	C_15_H_10_O_7_	302.0427	[M-H]^−^	301.03538	6.9	10.42	−3.405739	0.00671	–
12	Apigenin	C_15_H_10_O_5_	270.0528	[M-H]^−^	269.04555	7.1	11.59	4.657361	0.0009	+
13	ArjunglucosideII	C_36_H_58_O_10_	650.403	[M-H]^−^	649.39572	2.5	11.72	3.264763	0.00851	+
14	Ethyl 2-hydroxytetradecanoate	C_16_H_32_O_3_	272.2352	[M-H]^−^	271.22787	6.5	14	6.580312	6.23E-05	+
15	3,5-Di-tert-butyl-4-hydroxybenzaldehyde	C_15_H_22_O_2_	234.162	[M-H]^−^	233.1547	6.9	14.12	2.370131	0.03927	+
16	Maslinic acid	C_30_H_48_O_4_	472.3553	[M-H]^−^	471.34798	5.6	14.16	9.815299	1.89E-06	+
17	Diisobutyl phthalate	C_16_H_22_O_4_	278.1518	[M+H]^+^	279.15909	0.8	14.62	−8.161049	9.88E-06	–
18	9,12,15-Octadecatrienoic acid	C_18_H_30_O_2_	278.2246	[M+H]^+^	279.23186	0.8	15.77	3.118799	0.0109	+
19	Ethyl (9E,12E,15E)-octadeca-9,12,15-trienoate	C_20_H_34_O_2_	306.2559	[M+H]^+^	307.26316	−0.9	15.82	4.285773	0.0016	+
20	Ursolic acid	C_30_H_48_O_3_	456.3604	[M-H]^−^	455.35307	6.1	16.06	−2.962918	0.01422	–
21	Betulic acid	C_30_H_48_O_3_	456.3604	[M-H]^−^	455.35307	6.1	16.06	−2.962918	0.01422	–
22	Oleanolic acid	C_30_H_48_O_3_	456.3604	[M-H]^−^	455.35307	5.6	16.07	−2.962918	0.01422	–
23	Cistanoside H	C_16_H_30_O_2_	254.2246	[M-H]^−^	253.2173	5.7	16.27	4.714915	0.00082	+
24	Palmitoleic acid	C_16_H_30_O_2_	254.2246	[M-H]^−^	253.2173	3.9	16.27	4.714915	0.00082	+
25	Linoleic acid	C_18_H_32_O_2_	280.2402	[M-H]^−^	279.23295	7	16.54	10.40155	1.11E-06	+
26	Z,Z-10,12-Hexadecadien-1-ol acetate	C_18_H_32_O_2_	280.2402	[M-H]^−^	279.23295	7	16.54	10.40155	1.11E-06	+
27	Linoelaidic acid	C_18_H_32_O_2_	280.2402	[M-H]^−^	279.23295	4.6	16.55	10.40155	1.11E-06	+

To further excavate the differences in *C. officinalis* between R and HPWS products, the data were standardized with SIMCA 14.1 (version, country) and analyzed *via* PLS-DA under supervised recognition mode. The results in Figure 5 show the PLS-DA score plot of *C. officinalis* before and after processing. As can be seen, *C. officinalis* processed with R and HPWS s were obviously clustered into two categories, indicating that the processing has changed the chemical composition in *C. officinalis*.

The current results also showed that along with HPWS processing the chemical composition in *C. officinalis* changed qualitatively, including 5-HMF, linoelaidic acid, and quercetin; 5-HMF prevents L02 hepatocytes from injury induced by GalN/TNF-α (28) and attenuates liver fibrosis by inhibiting oxidative stress in mice (29). Furthermore, quercetin can protect the liver and kidney, as reported in another study (30). Thus, this research could reveal a chemical basis for the enhanced anti-fibrosis activity of *C. officinalis* processed with HPWS.

## Conclusions

As a kind of medicine and edible herbal, *C. officinalis* has been shown to suppress liver and kidney fibrosis by inhibiting the activation of HSC-T6 and HK-2 cells. Studies have also shown that its anti-fibrosis activity can change with processing. Based on this information, our experiment used enhanced anti-fibrosis effects as the indicator to optimize the processing technology of *C. officinalis* through single-factor and orthogonal tests. Finally, we identified the processing that can produce optimal anti-fibrosis activity, namely, *C. officinalis* with HPWS, whose chemical composition was identified by UHPLC-Q-TOF-MS/MS analysis. The experimental result is a further step. The results of this study also provided material basis for further exploring the role of *C. officinalis* in liver and kidney protection.

## Data Availability Statement

The original contributions presented in the study are included in the article/Supplementary Material, further inquiries can be directed to the corresponding author/s.

## Author Contributions

GC and PX contributed to the design of the study, acquisition of data, and analysis and interpretation of the data. XH, CD, and YN contributed to the acquisition of data and analysis and interpretation of data. All authors participated in drafting or revising the manuscript and approved the final version of the manuscript for submission.

## Funding

This work was financially supported by the National Natural Science Foundation of China (Nos. 81973481, 81922073, and 8210142131), the Traditional Chinese Medicine Key Scientific Research Fund Project of Zhejiang Province (No. 2018ZY004), and Zhejiang Province Traditional Chinese Medicine Science and Technology Program (2022ZQ033).

## Conflict of Interest

The authors declare that the research was conducted in the absence of any commercial or financial relationships that could be construed as a potential conflict of interest.

## Publisher's Note

All claims expressed in this article are solely those of the authors and do not necessarily represent those of their affiliated organizations, or those of the publisher, the editors and the reviewers. Any product that may be evaluated in this article, or claim that may be made by its manufacturer, is not guaranteed or endorsed by the publisher.

## Supplementary Material

Supplementary Figure 1 Anti-fibrotic activity of main ingredients in *C. officinalis* processed on HSC-T6.

Supplementary Table S1 *C. officinalis* with HPS.

Supplementary Table S2 Variance analysis of *C. officinalis* with HPS.

Supplementary Table S3 *C. officinalis* with WS.

Supplementary Table S4 Variance analysis of *C. officinalis* with WS.

Supplementary Table S5 *C. officinalis* with HPWS.

Supplementary Table S6 Variance analysis of *C. officinalis* with HPWS.

## Abbreviations

TCM: traditional Chinese medicine
5-HMF: 5-hydroxymethylfurfural
HUVECs: human umbilical vein endothelial cells
ECM: extracellular matrix
α-SMA: alpha smooth muscle actin
HK-2: human proximal tubular epithelial cell
TIF: tubulointerstitial fibrosis
HSC-T6: hepatic stellate cells
EMT: epithelial mesenchymal transition
TGF-β: transforming growth factor β
UHPLC-Q-TOF-MS/MS: ultra-high performance liquid chromatography coupled with hybrid triple quadrupole time-of-flight mass spectrometry
ChP: Pharmacopeia of the People's Republic of China
HPS: high-pressure steamed
HPWS: high-pressure wine steamed.
