# Supplementary figures and images for: Optimizing Processing Technology of Cornus officinalis: Based on Anti-Fibrotic Activity
Source: Front Nutr. 2022 May 3;9:807071. doi: 10.3389/fnut.2022.807071 (PMC9113564; doi:10.3389/fnut.2022.807071)

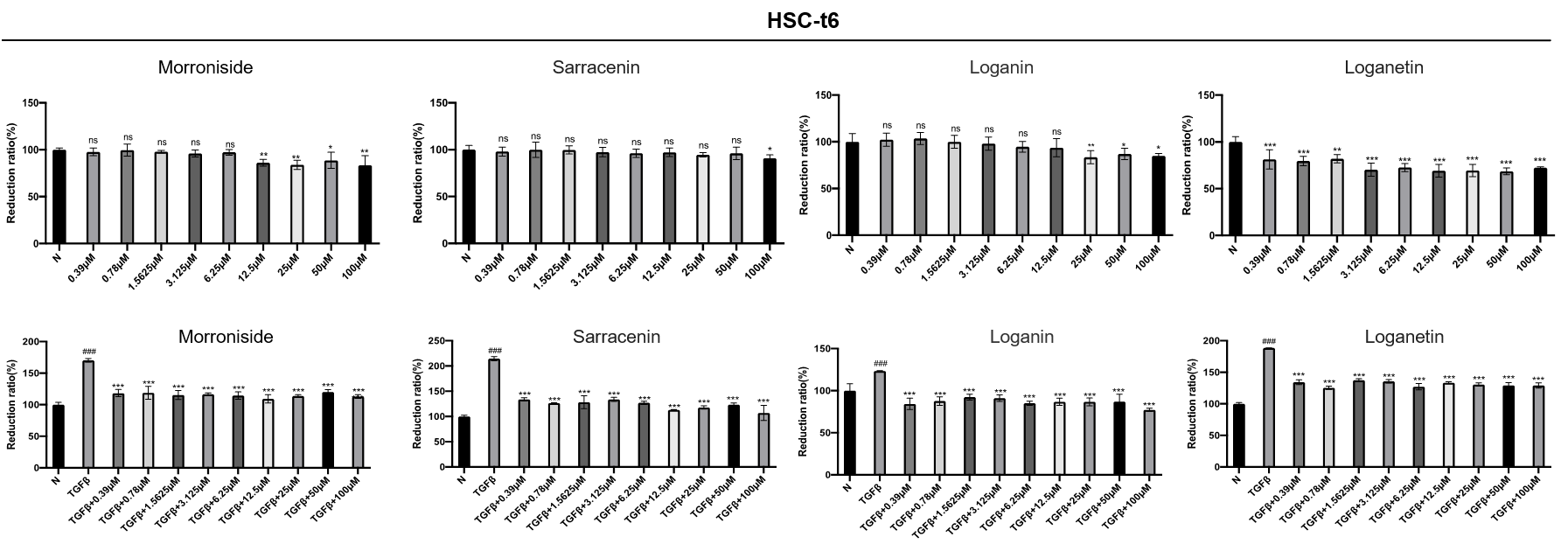

Supplement: Supplementary Figure 1 — Anti-fibrotic activity of main ingredients in C. officinalis processed on HSC-T6. [file Image_1.TIF]
