# Supplementary material for: Optimizing Processing Technology of Cornus officinalis: Based on Anti-Fibrotic Activity
Source: Front Nutr. 2022 May 3;9:807071. doi: 10.3389/fnut.2022.807071 (PMC9113564; doi:10.3389/fnut.2022.807071)
Supplement: Supplementary Table S1 — C. officinalis with HPS. [file Data_Sheet_1.PDF]

Supplementary Table S1: *C. officinalis* with HPS.

Supplementary Table S2: Variance analysis of *C. officinalis* with HPS.

Supplementary Table S3: *C. officinalis* with WS.

Supplementary Table S4: Variance analysis of *C. officinalis* with WS.

Supplementary Table S5: *C. officinalis* with HPWS.

Supplementary Table S5: Variance analysis of *C. officinalis* with HPWS.

Table S1 *C. officinalis* with HPS.

Number	Steam (°C)	Steam (h)	blank1	blank2	HK-2	HSC-T6	Composite score
1	105	1	-	-	1.84	2.00	1.92
2	105	2	-	-	1.00	1.44	1.22
3	105	3	-	-	1.40	1.47	1.43
4	115	1	-	-	1.94	1.59	1.77
5	115	2	-	-	1.53	1.11	1.32
6	115	3	-	-	1.21	1.00	1.10
7	125	1	-	-	1.55	1.32	1.43
8	125	2	-	-	1.92	1.86	1.89
9	125	3	-	-	1.37	1.28	1.33
K1	1.523	1.707	1.637	1.523			
K2	1.397	1.477	1.440	1.250			
K3	1.550	1.287	1.393	1.697			
R	0.153	0.420	0.244	0.447			

Table S2 Variance analysis of *C. officinalis* with HPS.

Error Source	SS	DOF	MS	F	P
Steam (℃)	0.040	2	0.020	0.199	0.827
Steam (h)	0.265	2	0.133	1.313	0.364
Error	0.404	4	0.101		

Table S3 *C. officinalis* with WS.

Number	Braise (h)	Wine (%)	Steam (h)	Blank	HK-2	HSC-T6	Composite score
1	0.5	25	1	-	1.97	1.98	1.97
2	0.5	30	2	-	1.68	1.73	1.70
3	0.5	35	3	-	1.33	1.38	1.36
4	1	25	2	-	1.89	1.62	1.76
5	1	30	3	-	1.75	1.42	1.58
6	1	35	1	-	1.48	1.22	1.35
7	1.5	25	3	-	1.02	1.00	1.01
8	1.5	30	1	-	1.44	1.68	1.56
9	1.5	35	2	-	1.50	1.97	1.73
K1	1.677	1.580	1.627	1.760			
K2	1.563	1.613	1.730	1.353			
K3	1.433	1.480	1.317	1.560			
R	0.244	0.133	0.413	0.470			

Table S4 Variance analysis of *C. officinalis* with WS.

Error Source	SS	DOF	MS	F	P
Braise (h)	0.089	2	0.044	0.359	0.736
Wine(%)	0.029	2	0.014	0.116	0.896
Steam (h)	0.278	2	0.139	1.119	0.472
Error	0.248	2	0.124		

Table S5 *C. officinalis* with HPWS.

Number	Braise (h)	Wine (%)	Steam (h)	Steam (°C)	HK-2	HSC-T6	Composite score
1	0.5	25	1	105	2.00	1.79	1.90
2	0.5	30	2	115	1.62	1.52	1.57
3	0.5	35	3	125	0.91	1.07	0.99
4	1	25	2	125	1.71	1.75	1.73
5	1	30	3	105	1.37	1.09	1.23
6	1	35	1	115	1.82	2.00	1.91
7	1.5	25	3	115	1.67	1.80	1.73
8	1.5	30	1	125	1.46	1.57	1.52
9	1.5	35	2	105	1.20	1.28	1.24
K1	1.543	1.767	1.760	1.523			
K2	1.643	1.510	1.567	1.733			
K3	1.557	1.467	1.417	1.487			
R	0.100	0.300	0.343	0.246			

Table S6 Variance analysis of *C. officinalis* with HPWS

Error Source	SS	DOF	MS	F	P
Wine (%)	0.289	2	0.145	8.304	0.107
Steam (h)	0.320	2	0.160	9.179	0.098
Steam (℃)	0.185	2	0.092	5.308	0.159
Braise (h)	0.035	2	0.017		
